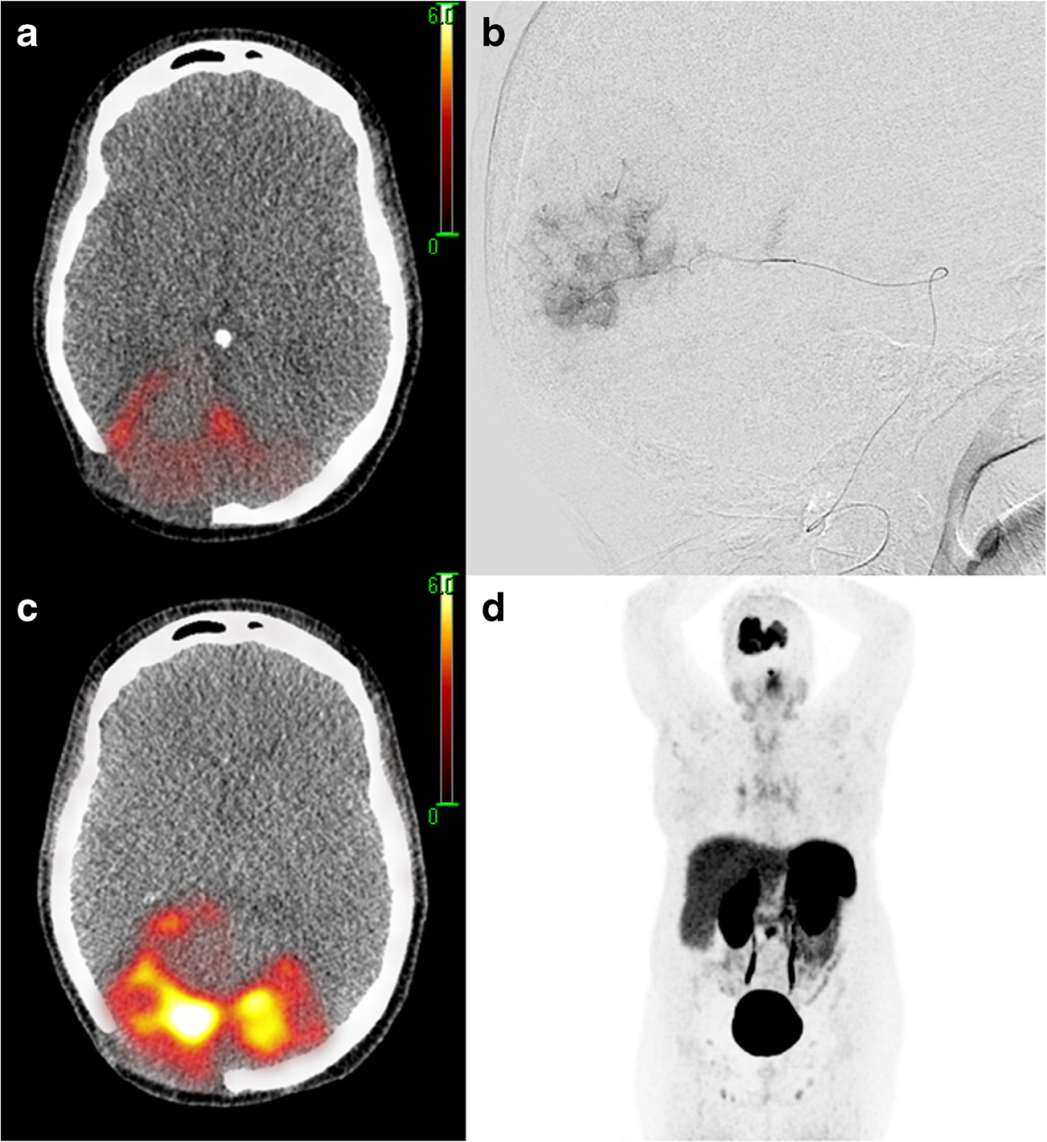# Intracranial hemangiopericytoma showing excellent uptake on arterial injection of [^68^Ga]DOTATATE

**DOI:** 10.1007/s00259-020-05096-z

**Published:** 2020-11-14

**Authors:** Sophie E. M. Veldhuijzen van Zanten, Eelke M. Bos, Frederik A. Verburg, Pieter-Jan van Doormaal

**Affiliations:** 1 Department of Radiology and Nuclear Medicine, Erasmus MC, Dr. Molewaterplein 40, 3015 GD Rotterdam, The Netherlands; 2 Department of Neurosurgery, Erasmus MC, Rotterdam, The Netherlands

A 40-year-old woman presented with a large intracranial tumour originating from the posterior cerebral falx. After careful evaluation by MRI and angiography, a meningioma was suspected and surgical removal was attempted. The procedure, however, was terminated prematurely due to profuse and uncontrollable bleeding from the tumour. Biopsies taken during surgery revealed a hemangiopericytoma. Radiotherapy was performed with 59.4 Gy, which caused tumour regression and local control for several years. Eventually, the patient progressed and was evaluated for possible peptide receptor radionuclide therapy (PRRT) using [^68^Ga]DOTATATE positron emission tomography/computed tomography (PET/CT), as previously it was described that hemangiopericytoma might show somatostatin receptor expression [1].

As after venous application [^68^Ga]DOTATATE uptake in the majority of the tumour did not exceed the uptake in the liver (i.e. Krenning score 2 [2], panel A), we decided to explore the possibility of increasing the uptake by injection in feeding arteries, as described previously [3–5]. Upon arterial injection in the posterior cerebral artery (Panel B), the mean lesional standardized uptake value increased from 8.4 to 21.0 and the maximum standardized uptake value from 15.8 to 36.0 (Panel C). As a result, the uptake in the entire tumour now exceeds the uptake in the liver (i.e. Krenning score 3, Panel D). This case shows that, especially with arterial application, PRRT can be considered as a serious therapeutic option in this rare disease entity. Our patient’s condition unfortunately deteriorated shortly after the diagnostic procedure to a point that therapy with [^177^Lu]DOTATATE was no longer possible.